# An annotation of cuts, depicted locations, and temporal progression in the motion picture "Forrest Gump"

**DOI:** 10.12688/f1000research.9536.1

**Published:** 2016-09-08

**Authors:** Christian O. Häusler, Michael Hanke

**Affiliations:** 1 Psychoinformatics Lab, Department of Psychology, University of Magdeburg, Magdeburg, Germany; 2 Center for Behavioral Brain Sciences, Magdeburg, Germany

**Keywords:** studyforrest, annotation, natural stimulation, scene perception, time perception, spatial cognition

## Abstract

Here we present an annotation of locations and temporal progression depicted in the movie “Forrest Gump”, as an addition to a large public functional brain imaging dataset ( http://studyforrest.org). The annotation provides information about the exact timing of each of the 870 shots, and the depicted location after every cut with a high, medium, and low level of abstraction. Additionally, four classes are used to distinguish the differences of the depicted time between shots. Each shot is also annotated regarding the type of location (interior/exterior) and time of day. This annotation enables further studies of visual perception, memory of locations, and the perception of time under conditions of real-life complexity using the studyforrest dataset.

## Introduction

Cognitive neuroimaging research is moving towards studying brain behavior under conditions of real-life-like complexity, and motion pictures are being utilized with increasing frequency as stimuli in “neurocinematics” studies ^1^. What sets motion pictures apart from other dynamic naturalistic stimuli is that they are more likely to evoke time-locked response patterns in a larger portion of the brain while retaining synchrony across multiple individuals who are experiencing the same movie ^2, 3^. One likely reason for this is the structure of movies. They are typically not prolonged, contiguous captures of an environment from a first person perspective, but rather they are carefully assembled, using “cuts”, from hundreds of short sequences shot from a variety of perspectives ^4^. These cuts are sharp discontinuities in the sensory input that require all viewers to re-assess the depicted environment in order to perform a cognitive re-orientation in fictional space and time. This re-orientation can be complex and involve a large bandwidth of cognitive processes: interpretation of contextual cues for detection of familiar settings, retrieval of prior knowledge from memory, discovery of change in locales and depicted characters. Consequently movies, and their cuts in particular, offer an excellent instrument to study complex, concurrent, real-life cognition.

In this study, we focus on spatial and temporal viewer re-orientation, and, to this end, describe changes in depicted location and time for all cuts in the motion picture “Forrest Gump”. This movie is the core stimulus of the *studyforrest* project ( http://studyforrest.org). Two fMRI datasets are publicly available: 1) participants listening to an audio-movie version ^5^ and 2) a subset of the original participants watching the audio-visual movie with simultaneous eye tracking ^6^. Additional imaging data and movie annotations are available ^7, 8^, including an individual localization of the *parahippocampal place area* ^9^ that has been implicated in spatial perception and scene processing ^10^.

This new annotation extends the available knowledge about the structure of this complex natural stimulus and enriches the overall studyforrest dataset. These data can be used to investigate the formation of a representation of viewer location and the perception of (speeded or negative) temporal progression in the movie stimulus. For any study focusing on other aspects of real-life cognition, these new data can serve as additional confound measures describing key properties of major building blocks of this movie stimulus.

## Materials and methods

### Stimulus

The annotated stimulus was a slightly shortened ( *≈*2 h) version of the movie Forrest Gump (R. Zemeckis, Paramount Pictures, 1994) with dubbed German soundtrack that is identical to the audio-visual movie annotated in 8. Further details on this particular movie cut, and how to reproduce it from commercially available sources, are available in 6.

**Table 1.** Example annotations for four shots at the beginning of the movie. Note that table headers do not literally correspond to column headers, see Data legend (ToD: time of day).

time	major location	setting	locale	int/ext	flow of time	ToD
300.56	Greenbow Alabama	doctor’s office	doctor’s office	ext	0	day
311.96	Greenbow Alabama	main street	crossroads	ext	+	day
318.28	United States	flashback countryside	flashback countryside	ext	-	day
343.04	Greenbow Alabama	main street	in front of barbershop	ext	++	day

### Annotation procedure

First, the movie was explored by two people, one of whom has an academic background in documentary film making, in order to generate a consistent list of labels for depicted and recurring locations.

Subsequently, the actual annotation was performed by the first author using a multi-pass strategy. The movie was manually inspected frame-by-frame to determine the location of cuts (using the video editor Shotcut v16.02.01). For each new shot (sequence between two cuts), a number of properties (described below) were discerned and entered into a table. A total of four passes were performed by the same observer in order to validate the annotation.

### Data legend

The annotation table contains one line per shot and seven columns: 1) a shot’s *start time*, 2) a label for the shot’s *major location*, 3) a label for the *setting* within the location, 4) a label for the *locale* within the setting, 5) a flag indicating an *interior or exterior* setting, 6) a label for the type of *temporal progression* with respect to the previous shot, and 7) a label for the *time of day*. Further details are provided in the following sections. The respective column header labels are given in parenthesis.

### Shot start time ( time)

A shot’s start time is defined as the onset time of the first video frame of a shot after a cut. Time stamps a provided in seconds of movie onset.

### Location

Location was coded with three labels, each describing the depicted scenery with an increasing level of detail.

**Major location** ( major_location) provides a coarse identification at the level of a town, county, or region where the respective story is taking place. Examples are: “Greenbow” or “Vietnam”.

**Setting** ( setting) further details the location by distinguishing places at the same major location, but are not in direct sight of each other. For example, Forrest Gump’s elementary school and the high school’s football field are both in Greenbow, Alabama but are not part of the same setting. A switch from one setting to another is typically synonymous with a transition to a new scene in a cinematographic sense. If the camera switched settings within a scene, the annotation deviates from the screenplay to make explicit the switch to another setting.

**Locale** ( locale) subdivides settings into distinguishable locales. Indoors, a locale is congruent with a particular room enclosed by walls. For example, Forrest Gump’s bedroom, the corridor downstairs, and the corridor upstairs are three different rooms inside the Gumps’ house (setting) on the Gumps’ property (major location). Outdoors, locales were distinguished when they were separated by a logical boundary, substantial distance, or shared no discernible landmarks. For example, the glade at the river and the location of the wounded Bubba are two different locales in the embattled jungle (setting) in Vietnam (major location). A locale’s label is identical to its setting label when only one locale is depicted for that setting.

### Interior or exterior ( int_or_ext)

This flag indicates whether a particular location is an open (“ ext”) or enclosed space (“ int”), such as a building or a vehicle.

### Temporal progression ( flow_of_time)

This label indicates the depicted progression of time between the previous and the current shot. Four categories were distinguished: “ -” labels a flashback, or jump into the past, independent of the temporal distance; “ 0” indicates no noticeable break in the ongoing stream of time, for example a sole change of viewing perspective; “ +” represents noticeable jumps in time, ranging from several seconds to about one or two hours; and lastly “ ++” marks major time jumps from several hours (e.g. night vs. day) to several years.

### Time of day ( time_of_day)

This flag indicates whether a scene is at least partially illuminated by sunlight. Consequently, daytime and twilight (early sunrises and late sun settings) are labeled as “ day”. If sunlight is entirely missing, the time of day is coded as “ night”.

### Dataset content

The released annotation is a single, text-based, comma-separated-value (CSV) formatted table ( Dataset 1).

CSV table with of depicted locations, and temporal progression in the motion picture “Forrest Gump” Each row corresponds to a shot in the the movie Click here for additional data file. Copyright: © 2016 Häusler CO and Hanke M 2016 Data associated with the article are available under the terms of the Creative Commons Zero "No rights reserved" data waiver (CC0 1.0 Public domain dedication).

The source code for all descriptive statistics included in this paper is available in Dataset 2 (Python script).

Python script to compute all descriptive statistics presented in the Data Note manuscript from the released annotations Click here for additional data file. Copyright: © 2016 Häusler CO and Hanke M 2016 Data associated with the article are available under the terms of the Creative Commons Zero "No rights reserved" data waiver (CC0 1.0 Public domain dedication).

## Dataset validation

To check for human error in the cut time annotation, timings were compared to the results of an automatic detection algorithm and any deviation was manually verified.

In summary, the shortened version of the movie comprises 870 shots (duration: min=0.48 s, max=151.08 s, median=4.92 s, SD=10.86 s). There are 612 shots depicting outdoor locations and 256 interior shots. Most shots take place during daytime (706 day vs. 162 night). The majority of cuts involve no noticeable discontinuities of depicted time (640), but there are 61 small and 135 large time jumps, as well as 32 flashbacks.

Table 2 provides information on the portrayal of unique locations in the movie.

**Table 2.** Descriptive statistics for all three levels of location annotation. *Number of shots* indicates the total number of shots in the movie for any particular location. *Number of consecutive shots* indicates how many shots are shown between two location changes at the respective level. *Times revisited* indicates how often a location reappears in the movie after it was depicted for the first time.

	major locations				settings				locales			
number of unique	21				95				147			
	min	med.	mean	max	min	med.	mean	max	min	med.	mean	max
number of shots	1	14	41.3	195	1	2	9.1	104	1	2	5.9	58
number of consecutive shots	1	2	7.3	70	1	2	4.7	69	1	1	3.2	32
times revisited	0	1	4.7	21	0	0	0.9	20	0	0	0.9	20

## Data and software availability

*F1000Research*: Dataset 1. CSV table with of depicted locations, and temporal progression in the motion picture “Forrest Gump”, 10.5256/f1000research.9536.d134823 ^11^

*F1000Research*: Dataset 2. Python script to compute all descriptive statistics presented in the Data Note manuscript from the released annotations, 10.5256/f1000research.9536.d134824 ^12^
