# Supplementary material for: An annotation of cuts, depicted locations, and temporal progression in the motion picture "Forrest Gump"
Source: F1000Res. 2016 Sep 8;5:2273. [Version 1] doi: 10.12688/f1000research.9536.1 (PMC5034791; doi:10.12688/f1000research.9536.1)
Supplement: Peer review history

# Peer review: Referee response for version 1

**DOI:** 10.5256/f1000research.10273.r16767

**Published:** 2016-10-03

**Authors:** Ming Meng

**Affiliations:** 1 Department of Psychological Brain Sciences, Dartmouth College, Hanover, NH, USA

This data note labels cuts in the motion picture "Forrest Gump" with starting time, three levels of location information, indoor/outdoor information, temporal progression, and the time of day. An automatic detection algorithm was also used to validate the labeling. It is very interesting as it provides an important tool for anyone who may be interested to study perception, attention, and other cognitive functions. I would like to thank the authors for their efforts and sharing this wealth of data.

 The introduction and data description including materials and methods are all clearly written. I approve this article.

 Minor points:

 It might be informative to provide to readers that among the total number of deviation of human labeling and automatic detection, how many were human errors and how many were computer errors.

 Page 3, last sentence: typos?

I have read this submission. I believe that I have an appropriate level of expertise to confirm that it is of an acceptable scientific standard.

# Peer review: Referee response for version 1

**DOI:** 10.5256/f1000research.10273.r16188

**Published:** 2016-09-22

**Authors:** Dylan Wagner

**Affiliations:** 1 Department of Psychology, Ohio State University, Columbus, OH, USA

This data note describes an annotation dataset consisting of time indices and annotations describing location and temporal information for each identified shot in the movie Forest Gump. This dataset of movie annotations is intended to be used with the associated functional neuroimaging datasets of participants viewing the movie Forrest Gump. All in all, I think this is an excellent addition to the growing empire that is StudyForrest, one that clearly represents an enormous amount of effort.

 I have only minor comments:

  

- The timing seems to be a bit off relative to the previously released dataset of scenes in the studyforrest GitHub repository. Presumably the annotation for shots and scenes should line up at scene starts but there appears to be consistent offset of about 12ms. For example, the last scene (“School bus stop”) starts at 6944.96 in the scenes.csv and 6944.84 in the attached dataset for shots in this paper. Moreover, the shots in this annotation don’t quite line up with the shots.csv on the github repo. The ~12ms offset is too large to be a single frame. It appears the authors switched from Advene to Shotcut for movie segmentation and annotation, perhaps therein lies the source of the mismatch?

 

- Could the authors expatiate on their method of identifying shots and cuts? If memory serves, in previous datasets they used an automated method to identify shots that was subsequently edited by hand. In this dataset, it appears all shots were identified by hand.  Where all cuts identified? Or are there special cases were two cuts appearing in close succession were considered a part of one shot? For instance, in an action heavy scene you could presumably get an overabundance of cuts, but that level of granularity isn’t really useful (nothing changes) and potentially these could be combined into a single shot. If every cut was indeed identified and annotated, then my sincere condolences to the coder!

 

- Although it is extremely generous of the authors to provide python code for generating descriptive data and associated figures, I’ve examined this code file and unfortunately this reviewer simply cannot support the premature use of Python 3… You can pry 2.7 from my cold dead hands. ;)

 

 Finally, I would like to again thank the authors for openly sharing this wealth of data with the community. These annotations and the associated imaging data represent a generous sharing of valuable resources, one that I have no doubt will be useful to many researchers interested in the neuroscience of naturalistic cognition.

I have read this submission. I believe that I have an appropriate level of expertise to confirm that it is of an acceptable scientific standard.

# Peer review: Referee response for version 1

**DOI:** 10.5256/f1000research.10273.r16187

**Published:** 2016-09-14

**Authors:** Julien Dubois

**Affiliations:** 1 Division of the Humanities and Social Sciences, California Institute of Technology, Pasadena, CA, USA; 2 Department of Neurosurgery, Cedars-Sinai Medical Center, Los Angeles, CA, USA

This Data Note presents a very useful (and labor intensive!) complement to the studyforrest dataset, providing additional annotations that can be used for data analysis: the timings of all the cuts in the movie, together with the depicted location and temporal progression for each transition. There are of course many, many other features that can be labeled in this movie, yet this particular set of features is useful on its own and will add to the bank of features already available. I have no major changes to suggest.

 Minor comments:

- would the authors consider publishing the code for the automated detection routine that they used to cross-check manual cut detection?
- a few cuts are annotated as locale "unknown_...". Is there no label that can be used rather than unknown? (I don't currently have the movie available to check these four cuts, at time stamps 4544.32, 4554.96, 4559.32 and 4561.56).

Very minor comments:

- the second sentence in the Introduction, "What sets motion pictures apart from other dynamic naturalistic stimuli is that they are more likely to evoke time-locked response patterns in a larger portion of the brain while retaining synchrony across multiple individuals who are experiencing the same movie", is rather unclear to me, in particular the use of "while" which indicates a contradiction -- why would time-locked responses in large portions of the brain not be synchronous across individuals?
- typos: 
  - in Materials and Methods/Shot start time (time) : correct to "Time stamps *are* provided in seconds *from* movie onset".
  - caption of Dataset 1: correct to "CSV table with depicted locations and temporal progression [...]"
- table 1: for consistency, maybe the column headers should be the same as in the actual csv file? 

I have read this submission. I believe that I have an appropriate level of expertise to confirm that it is of an acceptable scientific standard.
